# Genome-Scale Metabolic Modelling Approach to Understand the Metabolism of the Opportunistic Human Pathogen *Staphylococcus epidermidis* RP62A

**DOI:** 10.3390/metabo12020136

**Published:** 2022-02-02

**Authors:** Teresa Díaz Calvo, Noemi Tejera, Iain McNamara, Gemma C. Langridge, John Wain, Mark Poolman, Dipali Singh

**Affiliations:** 1 Quadram Institute Bioscience, Norwich Research Park, Norwich NR4 7UQ, UK; teresa.diaz-calvo@quadram.ac.uk; 2 Microbes in the Food Chain, Quadram Institute Bioscience, Norwich Research Park, Norwich NR4 7UQ, UK; noemi.tejera-hernandez@quadram.ac.uk (N.T.); gemma.langridge@quadram.ac.uk (G.C.L.); john.wain@quadram.ac.uk (J.W.); 3 Norwich Medical School, University of East Anglia, Norwich NR4 7UQ, UK; iainmcnamara@gmail.com; 4 Department of Orthopaedics and Trauma, Norfolk and Norwich University Hospital NHS Foundation Trust, Norwich NR4 7UY, UK; 5 Cell System Modelling Group, Oxford Brookes University, Oxford OX3 OBP, UK; mgpoolman@brookes.ac.uk

**Keywords:** non-aureus, *Staphylococcus epidermidis*, genome-scale metabolic model, linear programming, metabolic network, metabolism, auxotrophy, amino acids

## Abstract

*Staphylococcus epidermidis* is a common commensal of collagen-rich regions of the body, such as the skin, but also represents a threat to patients with medical implants (joints and heart), and to preterm babies. Far less studied than *Staphylococcus aureus*, the mechanisms behind this increasingly recognised pathogenicity are yet to be fully understood. Improving our knowledge of the metabolic processes that allow *S. epidermidis* to colonise different body sites is key to defining its pathogenic potential. Thus, we have constructed a fully curated, genome-scale metabolic model for *S. epidermidis* RP62A, and investigated its metabolic properties with a focus on substrate auxotrophies and its utilisation for energy and biomass production. Our results show that, although glucose is available in the medium, only a small portion of it enters the glycolytic pathways, whils most is utilised for the production of biofilm, storage and the structural components of biomass. Amino acids, proline, valine, alanine, glutamate and arginine, are preferred sources of energy and biomass production. In contrast to previous studies, we have shown that this strain has no real substrate auxotrophies, although removal of proline from the media has the highest impact on the model and the experimental growth characteristics. Further study is needed to determine the significance of proline, an abundant amino acid in collagen, in *S. epidermidis* colonisation.

## 1. Introduction

Staphylococci are non-motile Gram-positive cocci, and ubiquitous commensals of the skin and mucous membranes. They are opportunistic pathogens and the most frequent cause of nosocomial infections associated with implanted medical devices.

Although *Staphylococcus aureus* is probably the best-known and studied member of the genus, a number of non-aureus Staphylococci (NAS) are increasingly recognised as clinically significant pathogens [1,2,3]. Of particular concern, and the subject of this study, is *Staphylococcus epidermidis*, which can colonise numerous sites throughout the body and is now the most common NAS species associated with infections of indwelling medical devices, endocarditis and neonatal infections [2,4,5].

*S. epidermidis* is a facultative anaerobe and possesses complete Embden–Meyerhof–Parnas (glycolytic) and pentose phosphate pathways, as well as the tricarboxylic acid (TCA) cycle (which lacks the glyoxylate shunt) [6]. They mainly catabolise carbohydrates through the glycolysis and the pentose phosphate pathway, while the activity of the TCA cycle and electron transport chain depends on the availability of nutrients, as well as that of oxygen, nitrate or nitrite, all of which can serve as terminal electron acceptors [6,7,8].

The fact that *S. epidermidis* can survive in or on sites as diverse as skin, synovial fluid and the pericardium, suggests a great deal of flexibility in the utilisation of nutrient sources and this, in turn, implies considerable metabolic flexibility. Identifying the minimal nutrient requirements for growth and amino acid auxotrophies is a necessary first step in characterising this flexibility, although for staphylococci this has proven difficult [9,10,11] and, despite its clinical importance, there has been little or no study of the metabolic nutrient utilisation in *S. epidermidis*.

We propose that a better understanding of this flexibility would, in addition to the inherent contribution to basic science, have potential clinical applications, for example, in the development of prophylactic treatment against the colonisation of indwelling medical devices, in tailoring treatments to specific sites of infection, and in designing improved media for laboratory research [12].

Genome-scale metabolic models (GSMs) describe the network of reactions that are assumed to be present in a given organism, typically derived from an annotated genome, and may be used in a variety of endeavours, including the identification of novel pathways [13], metabolic engineering strategies [14], identification of potential drug targets [15,16], and the identification of minimal growth requirements (and design of minimal media) [17,18].

In this work, we have constructed the first curated genome-scale metabolic model for *S. epidermidis* and used it to investigate substrate preferences, and understand the growth requirements and potential routes of energy production in an aerobic environment. We have combined modelling and an experimental approach to investigate the metabolic abilities of *S. epidermidis* to utilise amino acids, its substrate auxotrophies and the impact of amino acid removal on growth and metabolism.

## 2. Materials and Methods

### 2.1. Model Construction

The model was constructed on the basis of the BioCyc Pathway/Genome Database (PGDB) [19,20] for *S. epidermidis* RP62A, version 20.1, using the ScrumPy metabolic modelling package [21] and in a modular fashion, using the approach described by Tejera et al. [18], Ahmad et al. [22], and was comprised of the following:

The top level module, which serves to import the modules listed below.
Automatically generated reactions extracted from the PGDB, automatically corrected where necessary, as described in Section 2.2.
Transport reactions to account for the import of the various media components and export of metabolic by-products (Section 2.4.1).
Biomass generation consisting of “pseudo-transporters” to allow for the export of biomass precursors. Biomass composition, comprised of biofilm and planktonic cell composition, was defined as a modification of that described for *S. aureus* [23] (Supplementary File SI).
Electron Transport Chain/Oxidative Phosphorylation in order to ensure correct stoichiometries for proton translocation, these reactions were defined in a manually generated module based on previous descriptions of staphylococci [24,25,26,27].
Additional reactions found to be necessary for the synthesis of biomass precursors, not present in the PGDB. Candidate reactions were included after confirming the presence of the genes encoding the corresponding enzymes or other experimental evidence in updated versions of BioCyc, the KEGG database for RP62A, and biochemical databases for *Staphylococcus* spp.

### 2.2. Model Curation and Theoretical Validation

Model curation and theoretical validation to ensure the stoichiometric and energetic consistency of the whole model was carried out using the approaches described in Tejera et al. [18], Gevorgyan et al. [28], Poolman et al. [29]. This is an iterative process: if an individual problem is identified (e.g., synthesis of ATP with no mass flow) modifications to individual reactions are corrected (e.g., by correcting the reversibility and/or directionality of a reaction) and the corrections are used to generate a new version of the model.

### 2.3. Model Analysis

#### Linear Programming Assumptions and Constraints

Model analysis was undertaken using linear programming (LP) with minimisation of total flux as the objective function. Additional constraints of cell maintenance cost, biomass production and substrate availability in the media were included to define the linear program as:

(1) $$\begin{array}{cc} \mathrm{minimise}: & \sum |v|\\ \;\\ \mathrm{subject}\;\mathrm{to}: & \left\{\begin{array}{c} N\cdot v=0\\ v_{i..j}=b_{i..j}\\ 0\leq v_{k..l}\leq m_{k..l}\\ v_{ATPase}=A \end{array}\right. \end{array}$$

where v is the vector of all reaction fluxes and N is the stoichiometry matrix; the objective is to minimise the sum of all (absolute) flux values (including transporters) based on the plausible assumption that cells tends to fulfil their functions at a minimal enzyme investment cost, as described in Holzhütter [30], Holzhütter [31], Singh et al. [32], $v_{i..j}=b_{i..j}$ defines flux in biomass transporters (μ× relative abundance), $v_{k..l}$ defines fluxes in the reactions importing media components with upper constraints determined by their relative abundance in the MHHW medium (described below and in Supplementary File SII).

$v_{ATPase}=A$ defines flux in a hypothetical ATPase reaction to account growth and non-growth-associated ATP demand, and is calculated as:

(2) $$A=Y_{ATP}\cdot \mu +m_{ATP}$$

where $Y_{ATP}$ and $m_{ATP}$ correspond to growth (60 mmol ATP gDW^−1^)- and non-growth (8 mmol ATP gDW^−1^ h^−1^)-associated maintenance cost, respectively [33,34]. μ value, calculated as described in Section 2.4.4.

The effect of removing individual amino acids from the medium was identified by setting a constraint of 0 to the associated transporter and setting μ in Equations (1) and (2) to the experimentally observed value (Section 3.3.2) when grown in its absence. The impact of the removal of the amino acid on the LP solution was calculated as the Euclidean distance of the solution’s flux vector from that of the solution calculated assuming the presence of all amino acids except glutamine (composition of defined rich media, as described in Section 2.4.1).

### 2.4. Experimental Conditions

#### 2.4.1. Defined Rich Media Design

Medium based on that described by Hussain et al. [35] (HHW medium) was modified by reducing the glucose concentration from 1% to 0.2%, as higher concentrations are known to induce biofilm formation [36,37] and adding asparagine; other compounds present in HHW medium that were previously reported as non-essential [38,39] were removed. The complete composition of this modified HHW medium (MHHW medium) media, containing all amino acids except glutamine, is described in Supplementary File SII.

#### 2.4.2. Inoculum and Bacterial Strains

The *S. epidermidis* RP62A strain used for experimental work was purchased from the National Culture and Type Collection (NCTC) and inocula were prepared as follows: a 25% glycerol stock was streaked out on BHI agar and incubated at 37 °C. After 24 h, 10 mL of fresh BHI broth were inoculated with 3 individual colonies and incubated for 18 h at 37 °C, shaking (180 rpm). Cells were recovered by centrifugation at 3000 rcf for 5 min. Finally, bacterial pellets were washed twice with sterile PBS, to minimise the potential carry over of nutrients, and re-suspended with PBS up to the original sample volume. An approximate concentration of 1.5 × 10^8^ CFU/ml for the bacterial inoculum was estimated by serial dilution and cell counting.

#### 2.4.3. Impact on Growth in the Removal of Individual Amino Acids

Amino acids were removed from the MHHW medium one at a time to determine the effect on growth characteristics. The reduction in total available C, N and S as a result of removing the amino acid was assumed to be negligible and no other adjustments were made to the composition of the medium. Original HHW medium and BHI were used as a positive control for growth.

Aliquots of inoculum were added in a 1/100 proportion to each test medium (20 μL to 2 mL), to give an approximate final concentration of 1.5 × 10^6^ CFU/mL. 150 μL of each sample was then added to three independent wells in a 96-well plate and incubated at 37 °C and shaking (180 rpm) for 48 h. Each experiment was performed in triplicate and cell growth was monitored by measuring absorbance spectrophotometrically at 600 nm (OD600) at 0, 4, 6, 8, 18, 24, 25, 26 and 48 h. No bacterial growth was observed when inoculated in an MHHW medium without any amino acids (minimal medium), indicating no potential nutrient carry-over. Non-inoculated MHHW medium broth and BHI broth were used as sterility controls and their OD measurements were used as background values, being subtracted from the absorbance measurements of the test samples at each timepoint.

#### 2.4.4. Growth Parameter Calculation

In order to make quantitative comparisons between experimental OD observations and model flux calculations, OD must first be converted into units of biomass (gDW.L^−1^). This was achieved by multiplying the OD value by a constant factor of 0.11, which was derived from preliminary experiments measuring OD and dry weight at the same timepoints.

As all inocula had reached stationary phase by 48 h, growth parameters were obtained by fitting the logistic (Verhulst’s) equation:

(3) $$y_{t}=\frac{y_{0}e^{\mu t}}{1+\frac{y_{0}}{y_{max}}\left(e^{\mu t}-1\right)}$$

where $y_{t}$ is the value of the population *y* at time *t*, $y_{max}$ is the maximum obtainable value of *y* (carrying capacity) and μ is the growth constant to the observed data. By setting $y_{t}=K/2$ and rearranging to make *t* the subject, we may also determine

(4) $$t_{0.5}=\frac{\ln(\frac{K-y_{0}}{y_{0}})}{\mu }$$

the time taken to reach half carrying capacity. In addition to being a slightly less abstract quantity than μ, this has the advantage of combining both of μ and K into a single value.

## 3. Results

### 3.1. Model: General Properties

The curated GSM of *S. epidermidis* RP62A consists of 895 reactions and 864 internal metabolites, with an additional 95 transport reactions and 74 external metabolites (Supplementary File SIII). All reactions are atomically balanced for carbon, nitrogen, phosphorous, sulphur, oxygen and hydrogen, and the model is also conserved for mass, energy and redox potential and free from stoichiometric inconsistencies. The general model properties are summarised in Table 1 and compared with those of other staphylococci available in the literature. The cellular overview diagram for *S. epidermidis* RP62A, generated through the Pathway Tools [40] version 23.0, is presented in Supplementary File SIV.

A total of 60 reactions not in the original PGDB were added during the process of model curation; these were mainly associated with the synthesis of biofilm, cell wall and cell membrane components. The remaining reactions were extracted from the PGDB, of which 611 reactions have the gene–protein-reaction (GPR) associations, 55 are spontaneous reactions, and the remaining reactions (169 reactions) have no gene–protein-reaction (GPR) associations and are inferred through the pathway prediction algorithm in the PGDB.

### 3.2. Model: Growth on MHHW Medium

The optimal solution to Equation (1), assuming that all components in the MHHW medium are available, contains 227 reactions, excluding transporters. Of these, 127 were found to be essential (i.e., the removal of any one of these results in the LP becoming insoluble).

**Figure 1:**
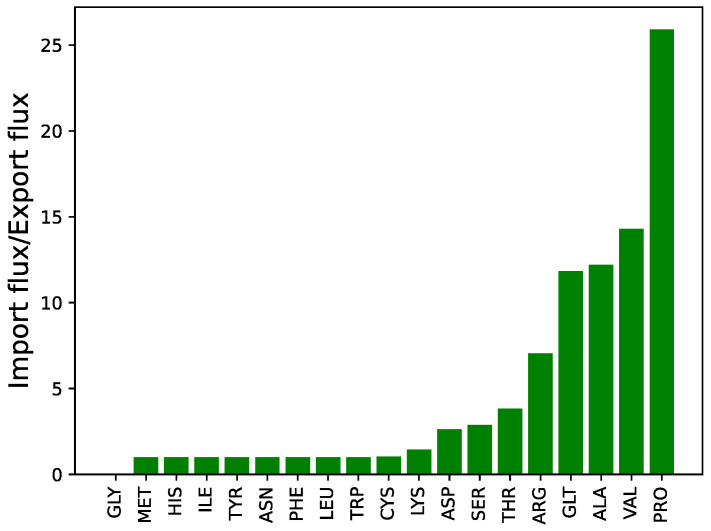
Ratio of flux in amino acid uptake transporters to their respective biomass exporters. Import flux of isoleucine, leucine, phenylalanine, tryptophan, tyrosine, asparagine, histidine and methionine is equal to the export of their respective biomass components. While the uptake flux for the remaining amino acids except glycine is higher than that in respective biomass exporters. There is no uptake of glycine from the medium and the demand for glycine in the biomass is met internally.

**Figure 2:**
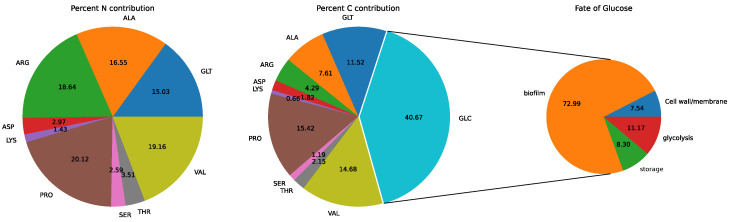
Percentage C and N contribution from the excess importation of amino acids in Figure 1. Among amino acids, proline, valine, alanine, glutamate and arginine are the major contributors in C and N. Although Glucose is the major contributor to C, only ≈10% enters glycolysis, while the remainder is utilised for the biofilm, storage and structural components of the biomass.

The import fluxes in isoleucine, leucine, phenylalanine, tryptophan, tyrosine, asparagine, histidine and methionine were equal to their respective biomass export fluxes, which is consistent with their direct incorporation into the biomass protein component and glycine, although its availability was not used. The uptake of amino acids relative to their biomass contribution is presented in Figure 1. The import of other amino acids exceeded that required to account for respective amino acid biomass components. The C and N contribution by the excess importation of these amino acids along with glucose is presented in Figure 2. This excess was greater than that required to account for the non-amino-acid biomass components, and excess C and N was excreted in the form of acetate and CO_2_, and NH_4_ respectively.

Examination of the LP solution (by manually tracing the major consuming and producing intermediate reactions, starting with glucose) showed that approximately 90% glucose consumption was used to satisfy the demand for biofilm, storage and structural components, with only 10% entering glycolysis.

**Figure 3:**
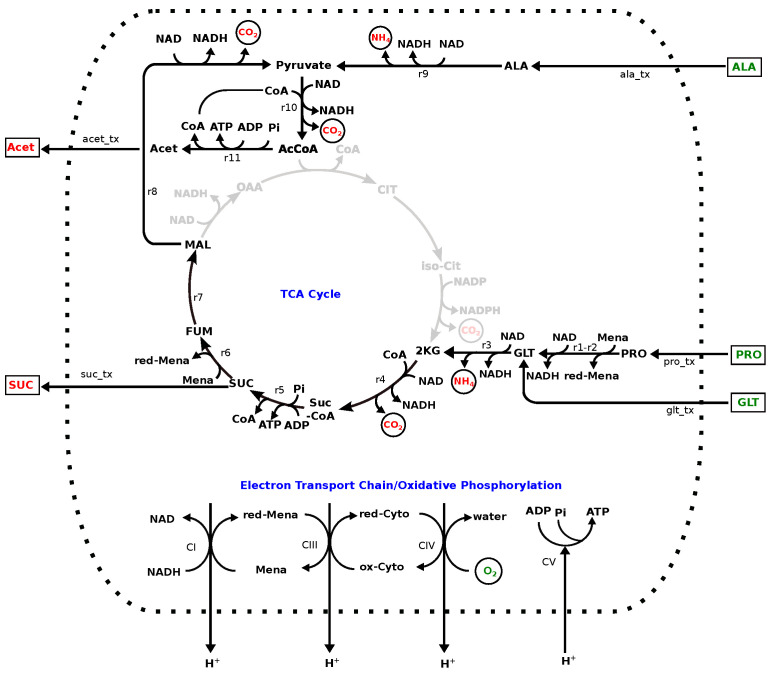
Linear programming solution with the constraint on ATPase flux, cell maintenance cost computed on MHHW medium, in the absence of demand for biomass precursors. All components of the MHHW medium, described above, were assumed to be present. Compounds in green (Pro, Ala, Glt, O_2_) are imported and those in red (Suc, Ac, CO_2_ and NH_4_) are exported. Reaction labels are r1: proline dehydrogenase, r2: 1-pyrroline-5-carboxylate dehydrogenase, r3: glutamate dehydrogenase, r4: 2-oxoglutarate dehydrogenase, r5: succinyl-CoA synthetase, r6: succinate dehydrogenase, r7: fumarate hydratase, r8: malate dehydrogenase, r9: alanine dehydrogenase, r10: pyruvate dehydrogenase, r11: acetate-CoA ligase, CI: NADH menaquinone reductase, CIII, menaquinol-cytochrome-c reductase, CIV: cytochrome-c oxidase, CV: ATP synthase.

One explanation for the excess amino acid uptake is that these compounds are catabolised for energy generation; in order to investigate this further, Equation (1) was solved in the absence of any demand for biomass, but with the ATP demand unchanged. The resulting solution has 26 reactions including the electron transport chain, some of the reactions associated with the TCA cycle and reactions involved with amino acid catabolism, as shown in Figure 3. It oxidises proline, alanine and glutamate as substrates and generates NH_4_, CO_2_, acetate and succinate as by-products. It is noteworthy that, although glucose was available, it was not utilised in this solution (see discussion).

### 3.3. Impact of Removing Individual Amino Acids

#### 3.3.1. Model

As described above, Equation (1) was repeatedly solved with individual amino acid transporters constrained to zero; no amino acids were found to be essential. The impact on the resulting flux distribution (in comparison to the MHHW solution) was determined as the Euclidean distance between the two flux vectors (Table 2). This has the advantage of accounting for changes in the distribution of reactions in utilised cases as well as the change in total flux in the system.

#### 3.3.2. Experimental

Optical density measurements were first converted to biomass concentration (gDW.L^−1^), and these were then used to determine the parameters μ, *K* and $t_{0.5}$, as presented in Table 3 (further details in Supplementary File SIV).

Growth in the absence of valine proved impossible to interpret: there was negligible but statistically significant growth (two-tailed *t*-test $p<10^{-4}$) until 30 h; between 30 and 48 h, the OD of 4 of the 9 samples increased by approximately two orders of magnitude, but the remainder was not significantly different (two-tailed *t*-test p>0.05) from the 30 h results. It was also not possible to determine meaningful growth parameters for these data and these results are not presented. The absence of proline also had a major impact on the growth characteristics. However, in contrast to the results in the absence of valine, the data were fully consistent with logistic characteristic growth.

Examination of Table 3 shows that the removal of individual amino acids from the medium consistently reduced μ with the possible exception of growth in the absence of aspartate. However, the effect on the carrying capacity, *K*, was much more varied and *K* actually increased (markedly so in the case of in the absence of threonine and leucine). The time taken to reach half-maximum density ($t_{0.5}$) was increased in all cases except again, possibly, in the absence of aspartate. The amino acid whose removal had the greatest impact on both μ and *K*, was proline, although the impact of its removal had a less marked effect on $t_{0.5}$.

### 3.4. Comparison of Experimental and Model Results

**Figure 4:**
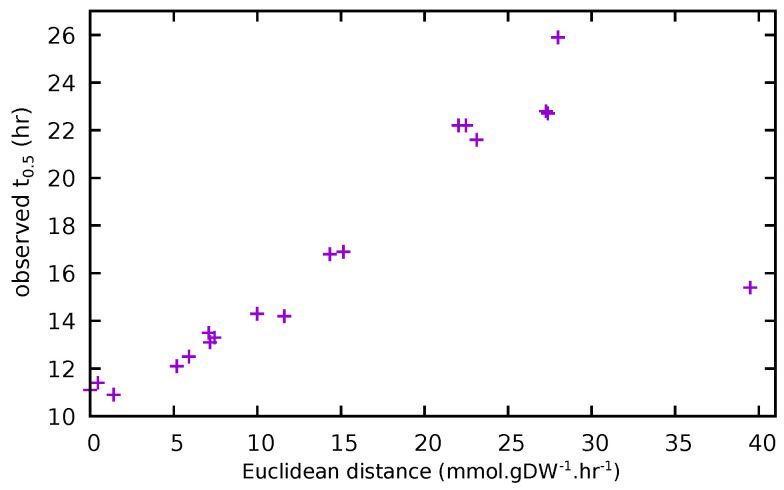
Comparison of calculated impacts of the removal of single amino acids (Euclidean distance) in the model and experimentally observed impacts ($t_{0.5}$). The outlying point at the lower right of the figure represents the -PRO medium.

As noted above, one advantage of determining $t_{0.5}$ from the growth data, in addition to being less abstract than μ, is that it integrates the two parameters, μ and *K*, into a single measurement. Similarly, when comparing steady-state flux vectors, determining the Euclidean distance simultaneously considers the changes in both the magnitude and direction of the flux vector. A comparison of these two methods of accounting for the impact of removing amino acids from the media, in vitro and in silico, is presented in Figure 4.

## 4. Discussion

Here, we present the first curated GSM of a non-aureus staphylococci, *S. epidermidis*. The properties of the model, in terms of number of reactions, transport processes, GPR relationships, etc., are compared to other published GSMs of *S. aureus* in Table 1. A more detailed functional comparison of the models can be found in Díaz Calvo [46]. Owing to the vast metabolic differences between *S. aureus* and *S. epidermidis*, we hope that this model will be of use to other investigators interested in studying *S. epidermidis* rather than utilising available *S. aureus* GSMs.

Although it is common to consider bacterial growth solely in terms of the constant μ, only considering exponential growth, in this study, growth properties were determined in terms of the logistic equation, which has the practical advantage of being able to utilise datapoints in the lag and stationary phases, as well as in exponential growth, and eliminate the potentially subjective identification of the start and end of the latter. Once fitted to the experimental data, it yields additional information in the form of the stationary phase cell density, *K*, as well as the growth constant, μ. This is relevant because, as we have shown (Table 3), alterations in media composition can have at least as much impact on *K* as upon μ.

**Figure 5:**
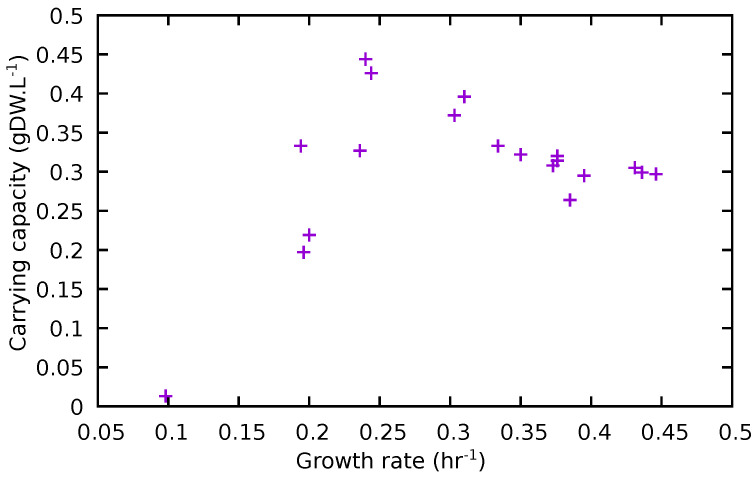
Relationship between calculated specific growth rate (μ) and carrying capacity (K).

Although no immediately obvious relationship appears to exist between μ and *K* in these data (Figure 5), over most of the observed range, an increase in μ leads to a decrease in *K*, suggesting a trade-off between rapid growth and the final attainable cell density. Whether or not this is related to nutrient depletion, the accumulation of specific metabolic by-products, or some other effect, cannot be determined on the basis of this dataset. Nonetheless, many studies of bacterial growth select conditions, and, indeed, very often strains, that are designed to maximise specific growth rate. However, as maximising the size of the final population would seem to be at least as reasonable a biological objective as maximising specific growth rate, it may be worth taking this into consideration in further laboratory studies. This leaves the question of what may be inferred about the behaviour of a pathogen in vivo, when neither the in vitro maximum specific growth rate or capacity are likely to be realised.

A third kinetic parameter, $t_{0.5}$, the time taken to reach half *K*, can also be calculated, and this has the advantage that, in addition to having a less abstract interpretation than μ, it can be readily estimated by inspection of the growth curve. More importantly, we have also shown that the impact of experimentally observed changes in the media upon $t_{0.5}$ can be directly related to the changes in calculated fluxes in the model, and serve to strengthen confidence in the subsequent interpretation of modelling results.

When a demand for biomass production was imposed in the model, assuming the presence of all proteogenic amino acids except glutamine in the medium (composition of MHHW medium), it is notable that they were not simply taken up in the proportions defined by the biomass composition, but varied considerably, between 0% in the case of glycine and 2590% in the case of proline, as shown in Figure 1. Valine, alanine, glutamate and arginine were other amino acids with high uptake proportions. Oxygen and glucose were also utilised, but only a small proportion (≈10%) of the latter entered glycolysis, with the remainder used as a precursor to biofilm, storage and structural components, as shown in Figure 2

It is interesting to note that, although the overall model solution utilised glucose and oxygen while excreting CO_2_, and that in an experimental setting such an observation would be taken as evidence for the operation of a conventional glycolysis/TCA/OP scheme, the model analysis suggests quite a different metabolic arrangement. This highlights the fact that a combination of modelling and experimental approaches makes it possible to identify potential modes of metabolism that could not be predicted on the basis of observation of input/output data alone.

In the absence of a demand for biomass, a fixed demand for ATP was satisfied utilising alanine, proline and glutamate as precursors, despite the presence of complete glycolytic and TCA cycle pathways and the availability of glucose. In the scheme described in Figure 3, proline and glutamate are oxidised via anaplerotic reactions to 2-oxoglutate, which is further oxidised to succinate and malate, the succinate is excreted and the malate further oxidised, via pyruvate to acetyl-CoA. However, this does not act as a substrate for citrate synthase to enter the TCA cycle, but is converted to acetate by acetyl-CoA-synthetase (ADP-forming) [47] with the formation of one molecule ATP, with acetate being exported. The synthesis of acetyl-CoA via pyruvate is also supported by the oxidation of alanine. In addition to providing some ATP by substrate-level phosphorylation the oxidation of these amino acids generates menaquinol, as well as NADH, as sources of reductant to the electron transport chain, which, in turn, utilises O_2_ as the terminal electron acceptor.

Similar schemes for the oxidation of amino acids to pyruvate and ultimately to acetate for ATP synthesis in staphylococci were previously described by a number of authors (e.g., Halsey et al. [11], Heinemann et al. [23], Tynecka et al. [26]), and the scheme presented here is generally consistent with that proposed by Halsey et al. [11] on the basis of a metabolomics study. It is perhaps not surprising that such closely related organisms should have similar metabolic processes, but it is interesting that these can be proposed on the basis of entirely different methodologies.

*Staphylococci* spp. have been reported to have varied amino acid auxotrophies [35,48,49,50], the most frequent being valine, leucine, cysteine, arginine and proline. However, more recent studies show that the latter two amino acids auxotrophies are condition-specific regulatory effects, and that carbon catabolite repression stops staphylococci growth in the absence of exogenous proline and arginine when glucose is present in the medium, rather than the lack of biosynthetic pathways [10,11,51]. Glycine auxotrophy has not been reported except in *S. epidermidis* small colony variants (slow-growing subpopulation of *S. epidermidis*) [52]. In this study, no amino acid auxotrophy was experimentally observed; model results were consistent with this and, as described previously, the absence of individual amino acids had varying but relatable impacts on experimental and calculated results.

A more detailed characterisation of the impact that these absences have upon flux distribution in the model, while both interesting and technically straightforward, is beyond the scope of the present investigation. The experimental observation that growth increased in the absence of certain amino acids is something that could not be explained from the model results. We propose, as a working hypothesis, that this effect results from changes in the concentration of signalling molecules in the environment and, as no simple LP method contains information about concentrations, this represents a fundamental limitation of the approach (although hybrid methods such as dynamic FBA [53] may offer a way forward). Nonetheless, as seen in Figure 4, the impact on the flux distribution in the model resulting from blocking individual amino acid uptake correlates well with experimental results.

## 5. Conclusions

The analysis of a strain-specific, genome-scale model of *S. epidermidis* RP62A has allowed us to investigate substrate requirements for growth in this strain. The results from this analysis suggested that, contrary to expectation, RP62A has no amino acid auxotrophies, and this was confirmed by subsequent experimental investigation. As part of the experimental work described here, we tested different iterations of a defined rich medium in which RP62A may be grown, and hope that this will be of use to other investigators requiring a defined growth medium and working with similar bacteria.

We have shown the potential for a complex interaction between amino acid and energy metabolism, whereby amino acids are catabolised with some substrate-level phosphorylation, generating pyruvate, which then feeds the electron transport chain and oxidative phosphorylation. Further experimental investigations are now needed in clinically relevant conditions to shed light on the factors that promote the colonisation of *S. epidermidis* in medical implants, and the ability of NAS to cause sepsis in neonates. In particular, the significance of proline utilisation in *S. epidermidis*, an amino acid present in high levels (10% of amino acids) in collagen, needs to be experimentally studied.

## Figures and Tables

**Table 1:** Comparison of model properties with previous *S. aureus* models. NR: Not reported; * excluding reactions with unspecified macromolecular formulae.

Strain (Publication)	Reactions	Transporters	Metabolites	Genes	Conservation	
					Mass	Energy
RP62A (this work)	895	95	864	611	yes	yes
iSA863 [41]	1545	NR	1379	NR	yes *	yes
iYS854 [42]	1440	NR	1327	NR	NR	yes
multiple [43]	1475	NR	1232	NR	NR	NR
multiple [44]	1497	146	1431	NR	NR	NR
iMH551 [23]	774	92	712	726	NR	yes
iSB619 [45]	640	84	571	581	no	NR

**Table 2:** Impact (ascending left to right) of inhibiting amino acid uptake on the model relative to MHHW. Impact is the Euclidean distance between the reference (MHHW) solution flux vector, and that obtained by inhibiting amino acid uptake.

Removed	Impact	Removed	Impact	Removed	Impact	Removed	Impact
None	0	ASN	0.462	ASP	1.41	SER	5.19
LYS	5.91	GLY	7.10	ILE	7.17	MET	7.44
CYS	10.0	HIS	11.6	TYR	14.3	PHE	15.2
THR	22.0	LEU	22.5	GLT	23.1	ARG	27.3
ALA	27.4	TRP	28.0	PRO	39.5	VAL	41.0

**Table 3:** Impact of removal of individual amino acids from the MHHW media on the growth characteristics of *S. epidermidis*: μ—specific growth rate, *K*—maximum biomass (gDW.L^−1^), *t*_0.5_—time (h) to reach 0.5 *K*.

Removed	μ	*K*	*t*_0.5_	Removed	μ	*K*	*t*_0.5_
None	0.436	0.299	11.1	ASN	0.431	0.305	11.4
ASP	0.446	0.297	10.9	SER	0.395	0.295	12.1
LYS	0.385	0.264	12.5	GLY	0.376	0.320	13.5
ILE	0.376	0.314	13.1	MET	0.373	0.308	13.3
CYS	0.350	0.322	14.3	HIS	0.334	0.333	14.2
TYR	0.310	0.396	16.8	PHE	0.303	0.372	16.9
THR	0.244	0.426	22.2	LEU	0.240	0.444	22.2
GLT	0.236	0.327	21.6	ARG	0.200	0.219	22.8
ALA	0.196	0.197	22.7	TRP	0.194	0.333	25.9
PRO	0.0982	0.013	15.4	VAL	ND	ND	ND

## Data Availability

The genome-scale metabolic model (in ScrumPy and SBML format) along with python scripts (version 3.0 and above) used in this study is available, on request, from https://gitlab.com/singhdi/staphylococcus-epidermidis-rp62a (accessed on 18 November 2021). GSM has also been deposited to the BioModels repository www.ebi.ac.uk/biomodels/ (accessed on 12 March 2021).

## Supplementary Materials

The following supporting information can be downloaded at: https://www.mdpi.com/article/10.3390/metabo12020136/s1, Supplementary File SI: Biomass composition used in the model, Supplementary File SII: Modified HHW media composition developed in this study, Supplementary File SIII: Genome-scale metabolic model of *S. epidermidis* RP62A, Supplementary File SIV: Cellular overview diagram of *S. epidermidis* RP62A. Supplementary File SV: Growth curve calculation.
