# Supplementary material for: Genome-Scale Metabolic Modelling Approach to Understand the Metabolism of the Opportunistic Human Pathogen Staphylococcus epidermidis RP62A
Source: Metabolites. 2022 Feb 2;12(2):136. doi: 10.3390/metabo12020136 (PMC8874387; doi:10.3390/metabo12020136)
Supplement: Supplementary file 1 [file CellularOverview_SupIV 2022.2.10.pdf › Supplementary/CellularOverview_SupIV 2022.2.10.pdf]

Sepi176279Cyc: *Staphylococcus epidermidis* RP62A Cellular Overview

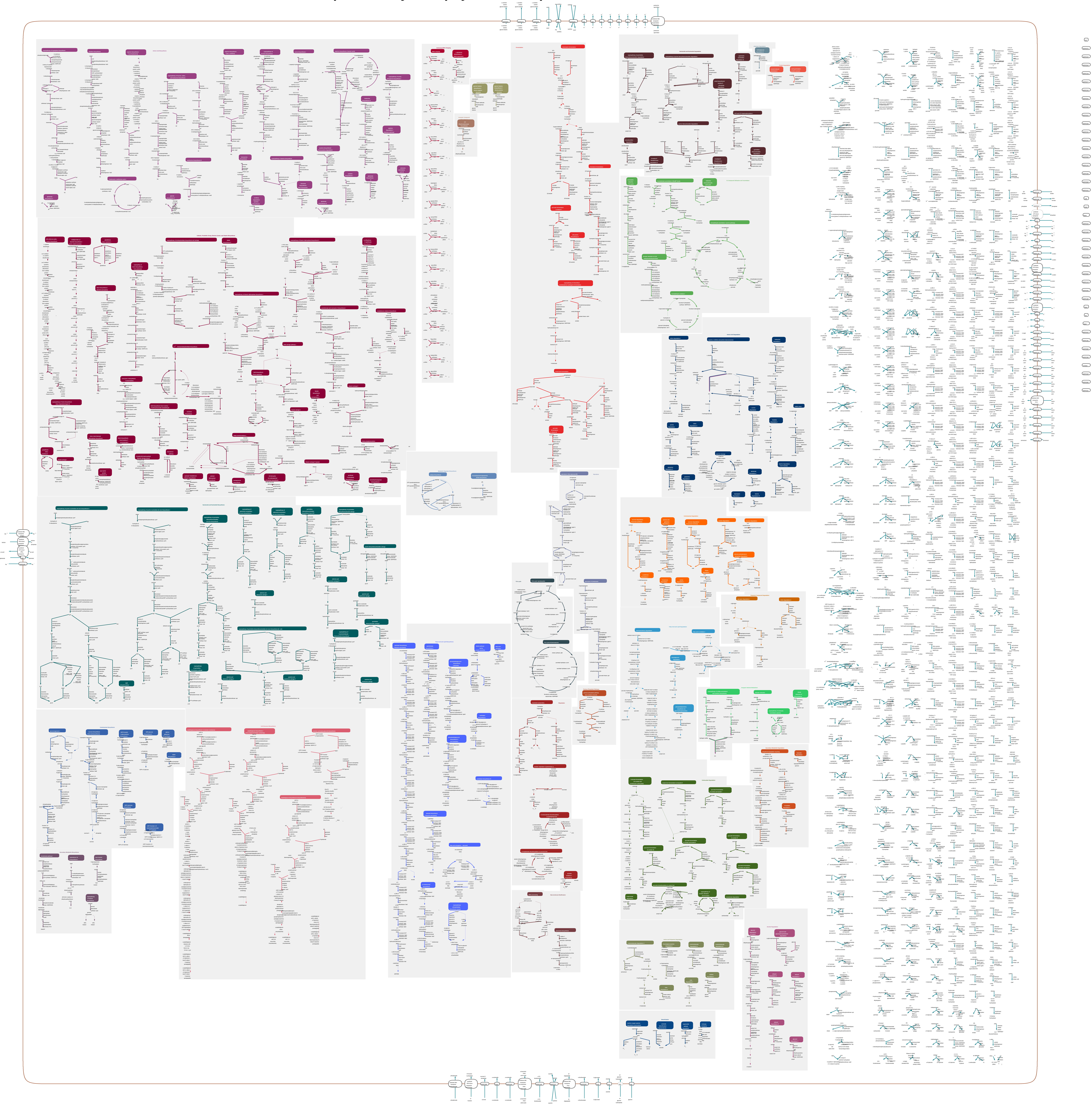

This diagram was automatically generated by SRI International Pathway Tools version 23.0, authors S. Paley and P.D. Karp.
