# Supplementary material for: Genome-Scale Metabolic Modelling Approach to Understand the Metabolism of the Opportunistic Human Pathogen Staphylococcus epidermidis RP62A
Source: Metabolites. 2022 Feb 2;12(2):136. doi: 10.3390/metabo12020136 (PMC8874387; doi:10.3390/metabo12020136)
Supplement: Supplementary file 1 [file metabolites-12-00136-s001.zip › Supplementary/BiomassComposition_SupI.pdf]

Table 1: Distribution of biomass precursors as described in the model. All values are mmol.GDW^{-1}. * Glycogen as abundance of glucose residues.

**AA residues**				**DNA**		**Biofilm**	
Ala	0.212	Leu	0.282	DATP	0.02	PIA1	3.345
Arg	0.111	Lys	0.235	DCTP	0.099	PIA2	1.110
Asn	0.173	Met	0.084	DGTP	0.099	PIA3	0.187
Asp	0.261	Phe	0.137	DTTP	0.02		
Cys	0.019	Pro	0.116	**RNA**			
Gln	0.2	Ser	0.198	ATP	0.061		
Glt	0.199	Thr	0.179	CTP	0.059		
Gly	0.19	Tyr	0.119	GTP	0.059		
His	0.073	Val	0.207	UTP	0.061		
Ile	0.269						

**Cell wall/membrane**		**Soluble metabolites**	
Diacylglycerol	0.019	Glycogen*	0.053
phosphatidylglycerol	0.045	AcCoA	36.5×10^{-6}
Cardiolipin	5.59×10^{-3}	SucCoA	2.21×10^{-6}
Lipoteichoic acid	3.35×10^{-3}	CoA	44.2×10^{-6}
diglucosyl-diacylglycerol	6.69×10^{-3}	FAD	74.1×10^{-6}
"3-DG-diacylglycerol"	1.12×10^{-3}	NAD	1.58×10^{-3}
Menaquinone	7.44×10^{-3}	NADH	36.5×10^{-6}
peptidoglycan	0.01	NADP	96.2×10^{-6}
Teichoic acid	0.062	NADPH	29.5×10^{-6}
