# Supplementary material for: Genome-Scale Metabolic Modelling Approach to Understand the Metabolism of the Opportunistic Human Pathogen Staphylococcus epidermidis RP62A
Source: Metabolites. 2022 Feb 2;12(2):136. doi: 10.3390/metabo12020136 (PMC8874387; doi:10.3390/metabo12020136)
Supplement: Supplementary file 1 [file metabolites-12-00136-s001.zip › Supplementary/GrowthCurves_SupV.pdf]

Fitting Growth Observations to the Logistic Function

February 1, 2022

Observations of optical density (3 technical replicates each of 3 biological replicates) were first converted to a biomass concentration (gDW^{-1}) by multiplying by the constant 0.11 which was derived from preliminary experiments measuring OD and dry weight at the same time points. The paramaters of the logistic equation

$$y_t = \frac{y_0 e^{\mu t}}{1 + \frac{y_0}{y_{max}}(e^{\mu t} - 1)} \quad (1)$$

y_0, μ and y_{max} (where y_t is the value of the population y at time t, y_{max} is the maximum obtainable value of y (carrying capacity) and μ is the growth constant) were determined using the `optimize.curve_fit(...)` function from the `scipy` software package

(https://docs.scipy.org/doc/scipy/reference/generated/scipy.optimize.curve_fit.html).

As it appeared that in some, but not all, of observations the bacteria fad entered death phase (declining OD) by 48 hrs, the 48 hr data was compared to the 30 hour data using a one-tailed t-test with the null hypothesis that the 48hr data was not lower than the 30 hr data. If this was rejected (p<0.05) 48hr data was included in the fitting process, otherwise not.

Two sets of results deserve additional comment: -PRO and -VAL. In the absence of proline growth was very slow, but nonetheless statistically significant. A one-tailed t-test with the null hypothesis that the 48 hr data was no greater than the 0hr data resulted in $p < 10^{-4}$.

The samples grown without valine yielded anomolous results, not rising above $5{\times}10^{-3}$ until 48 hrs when 4 of the 9 replicates rose to ≈ 0.3 $gDW.L^{-1}$ with the remainder remaining at ≈ 0.005 $gDW.L^{-1}$. The reason for this is unkown, but the standard deviations associated with the fitted paramaters for this data demonstrate them to be unreliable. They have been included here for completeness.

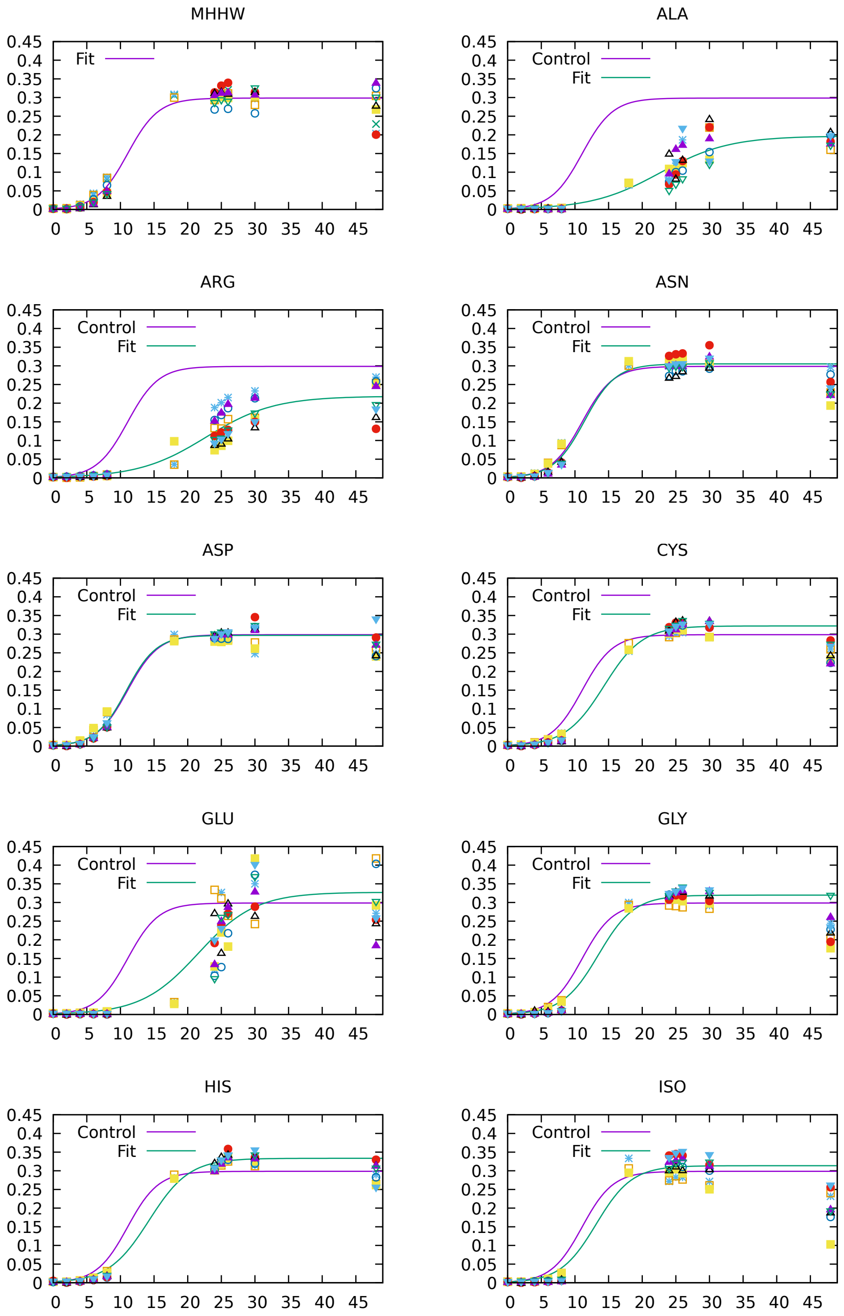

Figure 1: Growth characteristics of *S. epidermidis* in MHHW medium with the indicated amino acid removed. In all cases the x axis spans 0–48hrs and the y axis is labelled in units gDW.L^{-1}. Each symbol represents a replicate. The data are plotted along with the curve of the logistic function with parameters determined for that set along with the control - MHHW with no amino acids removed. The figure continues overleaf.

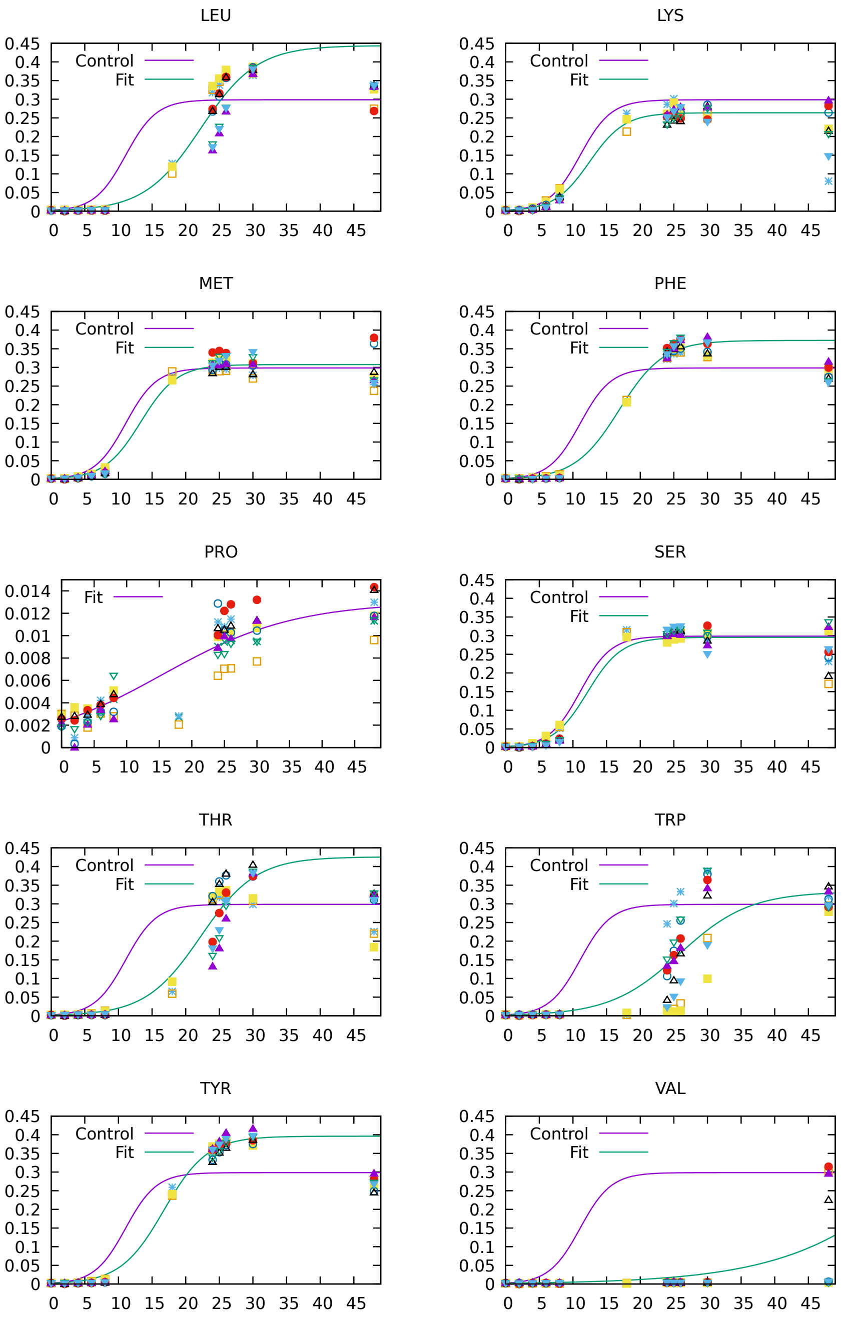

Figure 1: - continued.

Table 1: Growth parameters with the specified amino acid removed from the growth medium, MHHW is the control. Subscript σ denotes the standard deviation of the paramater estimates as calculated by the curve_fit(...) function.

	μ	μ_σ	y_{max}	y_{max_σ}
MHHW	0.436	0.071	0.299	3.46×10^{-3}
ALA	0.196	0.028	0.197	9.5×10^{-3}
ARG	0.2	0.028	0.219	9.63×10^{-3}
ASN	0.431	0.046	0.305	2.71×10^{-3}
ASP	0.446	0.047	0.297	2.44×10^{-3}
BHI	0.699	0.065	0.229	3.37×10^{-3}
CYS	0.35	0.015	0.322	2.41×10^{-3}
GLU	0.236	0.043	0.327	0.016
GLY	0.376	0.022	0.32	2.8×10^{-3}
HIS	0.334	0.015	0.333	2.93×10^{-3}
ISO	0.376	0.037	0.314	4.48×10^{-3}
LEU	0.24	0.032	0.444	0.031
LYS	0.385	0.029	0.264	2.52×10^{-3}
MET	0.373	0.034	0.308	3.76×10^{-3}
PHE	0.303	0.015	0.372	4.83×10^{-3}
PRO*	0.098	0.01	0.013	718×10^{-6}
SER	0.395	0.058	0.295	4.43×10^{-3}
THR	0.244	0.04	0.426	0.036
TRP	0.194	0.05	0.333	0.024
TYR	0.31	0.012	0.396	3.9×10^{-3}
VAL*	0.087	0.105	1	20.046
