# Supplementary material for: Genome-Scale Metabolic Modelling Approach to Understand the Metabolism of the Opportunistic Human Pathogen Staphylococcus epidermidis RP62A
Source: Metabolites. 2022 Feb 2;12(2):136. doi: 10.3390/metabo12020136 (PMC8874387; doi:10.3390/metabo12020136)
Supplement: Supplementary file 1 [file metabolites-12-00136-s001.zip › Supplementary/MHHWComposition_SupII.pdf]

Composition of the Modified HHW Media

Component	Concentration (mM)
Ammonium iron(II) sulfate hexahydrate($(NH_4^+)_2SO_4{\cdot}FeSO_4{\cdot}6H_2O$)	0.21
Calcium chloride anhydrous ($CaCl_2$)	0.45
Disodium hydrogen phosphate anhydrous (Na_2HPO_4)	56.1
Magnesium sulfate anhydrous ($MgSO_4$)	2.03
Manganese sulfate monohydrate ($MnSO_4{\cdot}\ H_2O$)	0.29
Potassium dihydrogen phosphate (KH_2PO_4)	22.0
Glucose	11.1
L-Ala	1.12
L-Arg	0.57
L-Asn	1.13
L-Asp	1.13
L-Cys	0.41
Gly	1.33
L-Glt	1.02
L-His	0.64
L-Ile	1.14
L-Leu	1.14
L-Lys	0.68
L-Met	0.67
L-Phe	0.60
L-Pro	1.30
L-Ser	0.95
L-Thr	1.26
L-Trp	0.49
L-Tyr	0.55
L-Val	1.28
Biotin	0.04
Nicotinic acid (niacin)	0.16
Thiamine hydrochloride	0.59

Final pH adjusted to 7.2 ± 0.2 at 25 using 0.1M NaOH.
